# Protocol for the “stand the future” trial: robotic exoskeleton gait training for non-ambulatory children with spastic cerebral palsy

**DOI:** 10.3389/fneur.2025.1651913

**Published:** 2025-10-15

**Authors:** Beier Xia, Na Mi, Zhixiang Wen, Yang Zhang

**Affiliations:** ^1^College of Sport Education, Shanghai University of Sport, Shanghai, China; ^2^College of Arts and Science, Hubei Normal University, Huangshi, China; ^3^College of Physical Education, Hunan Normal University, Changsha, China; ^4^Independent Person, Windermere, FL, United States

**Keywords:** fascicle length, muscle volume, motor development, gait, rehabilitation

## Abstract

**Purpose:**

Children with spastic cerebral palsy (CP) classified as Gross Motor Function Classification System (GMFCS) level IV face profound mobility limitations and are often excluded from intensive gait rehabilitation programs due to the severity of their impairments. Robotic-assisted gait training offers a promising avenue for functional improvement in this underserved population, yet empirical evidence remains scarce. This protocol describes a trial designed to evaluate the effects of a 6-month robotic gait training intervention on gross motor function, walking capacity, joint range of motion, muscle morphology, and psychological satisfaction in children with GMFCS level IV CP. Results will be reported in a subsequent publication following trial completion.

**Methods:**

This two-arm, parallel-group, randomized controlled trial will enroll 36 children aged 6–12 years with GMFCS level IV CP. Participants will be randomized 1:1 to either the intervention group (robotic gait training using the RoboCT Pediatric Lower Limb Rehabilitation Robot) or the control group (usual care as determined by family preference). The intervention group will receive 45-min sessions, three times per week for 24 weeks. Primary outcomes include Gross Motor Function Item Set and walking capacity (1-min walk test). Secondary outcomes include passive ankle joint range of motion, lower-limb muscle morphology via ultrasound, and psychological satisfaction. Linear mixed-effects models will evaluate group-by-time effects under an intention-to-treat framework.

**Discussion:**

This will be one of the first trials to explore long-term robotic gait training in children with GMFCS level IV CP. Findings may inform evidence-based rehabilitation approaches and improve access to technology-supported interventions in this highly underserved population.

**Clinical trial registration:**

ClinicalTrials.gov, NCT07049523.

## 1 Introduction

Cerebral palsy (CP) remains the most common motor disability in childhood, affecting approximately 17 million people worldwide, with an estimated 2–3 per 1,000 live births (1, 2). In China alone, the number of children with CP is estimated to exceed 6 million, with approximately 40,000 new cases occurring each year (3). Notably, a substantial proportion of these children are classified as Gross Motor Function Classification System (GMFCS) levels IV or V, indicating they are either non-ambulatory or severely limited in voluntary movement (4). Children with GMFCS levels IV and V often exhibit pronounced spasticity, limited voluntary muscle control, and severe deficits in selective motor control. Muscle fibers in these children show altered histological features including reduced cross-sectional area, increased collagen deposition, and impaired oxidative capacity (5). These pathological characteristics further exacerbate limitations in force generation and movement efficiency. These children experience profound impairments in mobility, postural control, and independence, presenting critical challenges for their physical and psychological development.

As we recently reported in a systematic review (6), current physical rehabilitation strategies for children with CP are derived from studies involving ambulatory children (GMFCS I–III), while the severely affected population (GMFCS IV–V) remains underrepresented in the literature. The absence of walking ability in these children precludes participation in many conventional physiotherapeutic or strength-based programs, resulting in stagnated motor development and a cascade of secondary complications including muscle atrophy, contractures, and social exclusion (7). In addition to clinical burdens, non-ambulatory children with CP present significant lifelong economic challenges for families and healthcare systems. Estimates suggest that the lifetime cost of care for a single individual with CP can exceed $1.6 million USD in high-income countries (8), with costs related to direct medical care, assistive equipment, caregiving, and loss of productivity. In China, the economic burden is compounded by disparities in access to rehabilitation services and limited insurance coverage for long-term care.

Recent advancements in robotics and wearable technologies have opened new avenues for restoring movement in neurologically impaired individuals. Over the past 5 years, pediatric exoskeleton systems have progressed considerably, with improvements in adaptive joint actuation, trajectory control, and real-time feedback mechanisms that enhance both safety and engagement (9). Robotic exoskeletons and powered orthoses offer the potential to provide task-specific, repetitive, and intensive gait training while minimizing therapist workload (10). Such devices have shown promise in adult populations with spinal cord injury and stroke (11), and early-stage trials have extended these technologies to pediatric neuromuscular disorders, including CP (12). However, empirical data on the feasibility, safety, and efficacy of prolonged robotic gait training in children with GMFCS IV and V classifications are virtually nonexistent.

For children with GMFCS level IV, the clinical goal of robotic-assisted gait training is not to achieve independent ambulation, but to preserve and enhance residual motor capacity. By enabling structured, repetitive upright movement, exoskeleton training may help maintain joint range of motion, stimulate muscle morphology, mitigate secondary complications such as contractures, and foster postural stability. In addition, the experience of supported upright mobility could promote psychosocial benefits, including motivation, engagement, and perceived quality of life. These outcomes, while modest compared with independent walking, are meaningful for improving comfort, health, and participation in daily life.

This protocol outlines a prospective 6-month intervention trial aimed at evaluating the effectiveness of robotic exoskeleton gait training on motor and psychological outcomes in children with severe CP. Through this study, we aim to establish initial empirical evidence for the feasibility and potential benefits of exoskeleton-assisted rehabilitation in children who are traditionally excluded from active gait training. We hypothesize that children with GMFCS level IV CP will demonstrate measurable improvements in supported motor function, joint flexibility, and lower-limb muscle morphology, as well as greater psychological satisfaction and therapy engagement. These expected benefits, though not equivalent to achieving independent gait, represent clinically relevant improvements that can reduce secondary complications and enhance quality of life. This trial addresses a major gap in pediatric neurorehabilitation and may provide a critical foundation for scaling up robotic interventions in underserved clinical populations.

## 2 Methods

### 2.1 Study design

**Figure 1:**
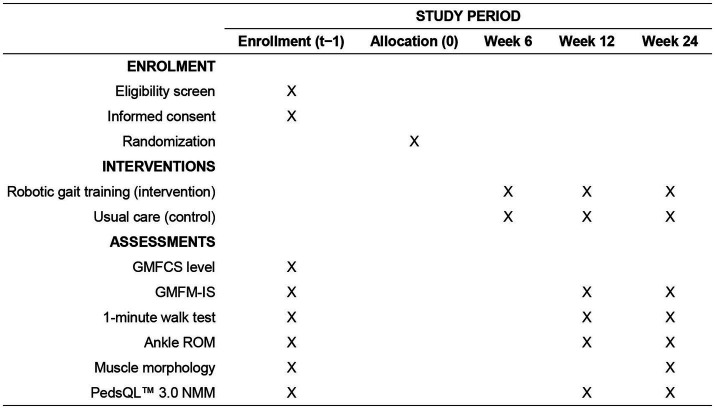
Schedule of enrolment, interventions, and assessments. GMFCS, Gross Motor Function Classification System; GMFM-IS, Gross Motor Function Measure – Item Set; PedsQL™ 3.0 NMM, Pediatric Quality of Life Inventory 3.0 Neuromuscular Module; ROM, range of motion.

This study is a prospective, two-arm, parallel-group, randomized controlled trial designed to evaluate the effectiveness of robotic-assisted gait training in children with spastic cerebral palsy classified as GMFCS level IV. The study will be conducted in the College of Physical Education at the Hunan Normal University, community rehabilitation centers and affiliated hospitals across the city of Changsha, Hunan Province, China. The trial follows the recommendations of the SPIRIT 2025 statement for standardized trial protocol reporting (13). As shown in Figure 1, all participants undergo eligibility screening and baseline assessments prior to randomization (week 0). The intervention phase spans 24 weeks, with robotic gait training or usual care delivered continuously across this period. Outcome assessments are scheduled at three timepoints: baseline (week 0), midpoint (week 12), and post-intervention (week 24), except for ultrasound imaging, which is conducted only at baseline and post-intervention. This structured timeline ensures standardized evaluation across participants and facilitates monitoring of both short- and longer-term effects of the intervention.

Participant recruitment is expected to begin in October 2025, with the intended recruitment period lasting approximately 3 months. Under the original plan, all eligible participants will be enrolled during this initial window, allowing for synchronized intervention delivery and outcome assessments. The overall study duration, including intervention and follow-up assessments, is projected to span 8 months, concluding by December 2026. However, in anticipation of potential delays in reaching the full target sample size during the initial recruitment phase, a contingency plan has been established to allow for phased enrollment. If fewer than the required 36 participants are enrolled by the end of the first recruitment window, a second recruitment phase will be conducted beginning no later than March 2026, with the aim of completing final enrollment by June 2026. In this scenario, intervention and assessment timelines will be adjusted accordingly, with outcome evaluations staggered by cohort while maintaining the same study protocol and follow-up durations. This dual-path recruitment strategy is designed to maximize enrollment feasibility while preserving methodological integrity and adherence to the trial timeline.

### 2.2 Trial status

This manuscript is based on Protocol Version 1.2, finalized in June 2025. At the time of manuscript submission, no participants have been enrolled. Recruitment is expected to begin in October 2025 and continue until June 2026, with the intervention and follow-up assessments scheduled through March 2027. The final report is expected to be published by June 2027.

### 2.3 Participants

Eligible participants will be children aged between 6 and 12 years with a clinical diagnosis of spastic CP, confirmed by a licensed pediatric neurologist from the Xiangya Hospital, Central South University. Only children classified as level IV on the GMFCS will be included. These children must rely on assistive mobility devices, such as wheelchairs or posture support walkers, for activities of daily living prior to enrollment. All participants must be medically stable, able to sit upright for at least 30 min, and capable of following simple verbal or visual instructions with caregiver support.

Children classified as GMFCS level V will not be eligible for inclusion, based on pre-study consultation with the device manufacturer and experienced pediatric physical therapists, who jointly determined that the functional and cognitive demands of the robotic exoskeleton used in this study would likely exceed the safe participation threshold for this population.

Exclusion criteria include prior administration of botulinum toxin-A injections to the lower limb muscles within 6 months prior to baseline assessment; the presence of an intrathecal baclofen pump; or a history of lower limb orthopedic or neurosurgical interventions, such as tendon lengthening or selective dorsal rhizotomy, at any time prior to study participation. Additionally, children will be excluded if they present with uncontrolled epilepsy, severe cognitive impairment, or other comorbid conditions that, in the judgment of the study physician or intervention team, may interfere with safe use of the robotic device or adherence to the study protocol.

Written informed consent will be obtained from each participant’s legal guardian prior to enrollment. When appropriate, verbal or pictorial assent will also be sought from the child.

### 2.4 Sample size

As of the trial planning period in 2025, no randomized controlled trial data are available investigating the effects of robotic-assisted gait training on both gait dynamics and lower-limb muscle function in children with spastic CP classified as GMFCS level IV. Due to the absence of prior randomized controlled trials in children with GMFCS level IV, the effect size for this trial was conservatively estimated based on findings from systematic reviews and meta-analyses in ambulatory children with milder CP (GMFCS I–III). While this introduces some uncertainty, it represents a standard methodological approach in feasibility trials where direct data are unavailable. Reported effect sizes from these studies are consistently large, with Cohen’s d values ranging from 0.8 for gait speed (14) to 1.65 for step length (15) improvements.

Considering the intensive, device-assisted nature of the intervention and the magnitude of improvement observed in related populations, an effect size of Cohen’s d = 1.0 was deemed appropriate for the sample size estimation in this trial. Using G*Power 3.1 software to model a two-tailed independent samples *t*-test with a significance level of 0.05, power of 0.80, and equal group allocation (1:1 ratio), a total of 34 participants (17 per group) were required to detect the hypothesized between-group differences. To accommodate a potential 5% dropout rate, the final recruitment target has been adjusted to 36 participants, ensuring adequate power to detect meaningful effects while accounting for minimal attrition.

### 2.5 Randomization and blinding

Participants will be randomly assigned in a 1:1 ratio to either the intervention group (robotic gait training) or the control group (usual care) using a computer-generated randomization sequence. The allocation sequence will be generated using a random number generator in R (package randomizr version 1.0.0) to ensure allocation concealment. Block randomization with a fixed block size will be employed to maintain balance between the groups throughout the recruitment period.

The assignment will be managed by a researcher not involved in participant enrollment or outcome assessment. Group allocation will be revealed to participants and caregivers following enrollment due to the nature of the intervention, which precludes participant-level blinding. This trial will follow a single-blind design, in which the statistician performing the primary and secondary analyses will remain blinded to group allocation until all data collection and preliminary data checks are complete. All outcome assessors collecting GMFCS scores, gait parameters, ultrasound muscle measures, and psychological questionnaires will remain blinded to group allocation to minimize detection bias. In the event that unintentional unblinding occurs, this will be documented, and an independent statistician—who is not otherwise involved in the trial—will be invited to conduct additional sensitivity analyses to ensure the robustness of the findings.

### 2.6 Intervention

The intervention group will receive robotic-assisted gait training using the RoboCT Pediatric Lower Limb Rehabilitation Robot (RoboCT Limited Co., Hangzhou, China), a pediatric exoskeleton system designed for overground locomotor training. This device integrates real-time motion sensing, adaptive control algorithms, and customizable joint actuation to deliver precise and individualized gait training for children with minimal voluntary motor control. The system includes powered actuators at the hip, knee, and ankle joints, a dynamic body-weight support harness, and multi-axis load and torque sensors for continuous monitoring of movement safety. Trajectory control is achieved through programmable gait cycles, allowing therapists to adjust joint angles, step length, and walking speed according to each child’s tolerance and progression. The device employs closed-loop feedback to ensure smooth movement execution and adaptive support. Safety features include automatic shutdown in response to excessive joint torque, misalignment detection, and redundant harness support. These integrated mechanical and control features ensure that the system can safely deliver task-specific, repetitive gait training in a pediatric population.

Robotic sessions will be conducted three times per week over a period of 24 weeks, with each session lasting approximately 45 min. All sessions will be conducted at a designated training site under the supervision of certified pediatric rehabilitation therapists. Each robotic training session will follow a structured protocol comprising several core components designed to promote safe, progressive, and task-specific gait rehabilitation. Sessions will begin with a brief warm-up period, which includes passive joint mobilization and postural alignment to prepare the child for exoskeletal engagement. Following this, participants will engage in assisted overground walking guided by the device’s programmable gait cycle, which delivers coordinated joint movements to simulate a physiological walking pattern.

Throughout the session, dynamic weight-shifting mechanisms will be activated to replicate the loading and unloading phases of gait, encouraging proprioceptive stimulation and trunk engagement. The system will allow for incremental adjustment of walking speed and step length, calibrated weekly to match each child’s individual tolerance and therapeutic progression. In parallel, integrated posture correction and trunk stabilization features will ensure proper alignment and mitigate compensatory strategies during walking.

To enhance engagement and maintain motivation, each session will incorporate a gamified feedback interface that provides real-time visual cues and positive reinforcement. These interactive elements are tailored to pediatric users and are intended to promote adherence through a rewarding, game-like experience. Load-sensing and joint torque feedback systems will continuously monitor safety and fatigue levels, enabling the exoskeleton to adjust support levels as needed. All session data will be digitally recorded to monitor progress and adjust programming parameters weekly.

Participants assigned to the control group will continue their usual care as determined by their caregivers, reflecting real-world rehabilitation practices in China. Usual care may include a combination of home-based stretching, passive range-of-motion exercises, school-based physiotherapy, or conventional outpatient therapy. To enhance comparability, all components of usual care (type, frequency, and duration) will be systematically documented at baseline and monitored during monthly check-ins. Analyses will include sensitivity and subgroup comparisons according to the type and intensity of usual care received. This approach allows us to preserve ecological validity while ensuring that outcome differences are not solely attributable to a weak or poorly defined comparator condition.

No co-interventions or crossovers between groups will be permitted during the intervention phase. Any adverse events, safety concerns, or deviations from the planned robotic training sessions will be logged and reviewed by the clinical monitoring team.

### 2.7 Outcomes

The primary outcome of this study will be gross motor function and walking capacity, assessed at baseline, middle-point, and after the 6-month intervention period. Secondary outcomes will include passive ankle joint range of motion, muscle morphological properties, and psychological satisfaction. All assessments will be conducted by trained evaluators blinded to group allocation.

Gross motor function will be evaluated using the Gross Motor Function Measure–Item Set (GMFM-IS), a validated short-form tool derived from the full GMFM-66, appropriate for assessing changes in children with CP (16). It focuses on key motor tasks across five dimensions: lying and rolling, sitting, crawling and kneeling, standing, and walking, running, and jumping. In this study, trained evaluators will observe children as they perform selected motor tasks from the GMFM-IS. Each task will be scored on a 4-point ordinal scale: 0 = does not initiate; 1 = initiates (<10% of the task); 2 = partially completes (10–99%); 3 = completes the task independently. The assessment will take place in a quiet, controlled environment with standardized instructions and safety precautions. Scores will be entered into the Gross Motor Ability Estimator software to generate interval-level total scores, allowing for sensitive tracking of changes in motor function over time.

To assess functional walking capacity, participants will undergo the 1-min walk test, performed on a marked 20-meter indoor course. Children will be instructed to walk as quickly and safely as possible without running, and the total distance covered in 60 s will be recorded. For participants who require orthoses or walkers, such assistive devices will be permitted during testing, consistent with real-world functionality.

Passive ankle joint range of motion (ROM) will be measured using an isokinetic dynamometer. The ankle was selected as the primary joint of interest because equinus contracture and limited dorsiflexion represent the most prevalent and clinically consequential deformities in children with GMFCS level IV CP. Restricted ankle mobility directly impacts orthosis fitting, postural alignment, and the ability to engage in supported upright activity. While contractures at the knee and hip are also common, reliable standardized measurement of these joints in this severely impaired population poses methodological challenges and would substantially increase participant burden. For these reasons, ankle ROM was prioritized as a sensitive, feasible, and clinically meaningful outcome measure. Each child will be seated with the knee fully extended, the ankle joint aligned with the dynamometer’s rotational axis, and the foot securely fixed to a footplate. Passive dorsiflexion and plantar flexion will be conducted through full available range to determine ROM and passive torque.

Muscle morphological properties of the lower limbs will be assessed via ultrasonography using a 6–12 MHz linear transducer (Meinianda BX-5, Zibo, China). Images will be acquired for the rectus femoris, quadriceps femoris, and medial gastrocnemius on both lower limbs. Standardized anatomical landmarks will be used for probe placement, and muscle thickness and muscle fascicle length will be quantified offline by blinded assessors.

Psychological satisfaction and treatment engagement will be evaluated using the Pediatric Quality of Life Inventory (PedsQL™ 3.0 NMM), which is validated for children with chronic motor impairments (17). This instrument includes child self-report and parent-proxy versions and assesses multiple dimensions including physical functioning, communication, and emotional well-being. Higher scores represent better perceived quality of life and satisfaction with daily function.

All outcome assessments will be performed at baseline (week 0), mid-point (week 12), and post-intervention (week 24), except for ultrasound imaging, which will be performed only at pre- and post-intervention timepoints.

### 2.8 Data management

Standardized case report forms will be used to record participant data in both paper and electronic formats. Data will be entered into a secure, password-protected database by trained research assistants and verified by a second reviewer to ensure accuracy. Each participant will be assigned a unique identifier to maintain confidentiality, and only de-identified data will be used for statistical analyses.

All data will be checked for completeness at each assessment point. In the case of missing data, a detailed log will be kept to identify the reasons and patterns of loss. If the amount of missing data exceeds 5%, multiple imputation methods will be applied under the assumption that data are missing at random. Sensitivity analyses will be conducted to assess the robustness of findings across imputed and complete-case datasets.

Descriptive statistics will be calculated for all variables to summarize baseline characteristics and monitor participant flow throughout the trial. Continuous variables will be reported as means ± standard deviations (or medians and interquartile ranges if non-normally distributed), and categorical variables will be presented as frequencies and percentages. Between-group differences at baseline will be tested using independent samples *t*-tests, Mann–Whitney U tests, or chi-square tests, as appropriate.

The primary analysis will follow the intention-to-treat principle, including all randomized participants regardless of adherence or dropout status. A repeated measures ANOVA or linear mixed-effects model will be used to evaluate between-group differences in changes over time for the primary outcomes (GMFM-IS scores and 1-min walk test distance). These models will include group (intervention vs. control), time (baseline, week 12, week 24), and a group-by-time interaction term. Subject-level random effects will account for within-participant correlations.

Exploratory subgroup analyses will be performed to examine the potential moderating effects of age group (6–8 years vs. 9–12 years), sex (male vs. female), and initial GMFM-IS baseline scores. These analyses will use interaction terms in the mixed-effects models to assess whether treatment effects differ across strata. Results will be interpreted with caution due to potential reductions in statistical power and the exploratory nature of these comparisons.

All statistical analyses will be performed using R, with a two-tailed alpha level of 0.05 considered statistically significant. A detailed statistical analysis plan will be finalized prior to database lock.

### 2.9 Ethics and dissemination

This study protocol has been reviewed and approved by the Ethics Committee of Hunan Normal University (approval number: 2025–506) and will be conducted in accordance with the ethical principles outlined in the Declaration of Helsinki. The trial protocol was prospectively registered in the ClinicalTrials.gov under registration number NCT07049523, dated July 2, 2025.

The intervention poses minimal risk to participants. The robotic exoskeleton system is clinically approved for pediatric rehabilitation and is equipped with comprehensive safety mechanisms, including load-sensitive shutdowns and postural supports. All sessions will be supervised by trained rehabilitation specialists to ensure safe operation. Participants in the intervention group may experience temporary fatigue or discomfort, but serious adverse effects are not anticipated. The potential benefits include improved gait performance, joint mobility, and psychological well-being in children with GMFCS level IV CP, a group that currently lacks access to high-intensity ambulatory interventions. Participants in the control group will not be restricted from pursuing usual care.

All personal data collected during the study will be handled in strict confidence. Participants will be assigned anonymized identification codes, and any linkage to identifiable information will be securely stored and accessible only to the principal investigators. Paper forms will be stored in locked cabinets, and electronic data will be stored on encrypted, password-protected servers. Data access will be limited to authorized research personnel. Final datasets used for statistical analysis will be fully de-identified.

The results of this trial will be disseminated regardless of the direction or statistical significance of findings. Findings will be submitted for publication in peer-reviewed, open-access journals specializing in pediatric rehabilitation, neurodevelopmental disorders, or assistive technologies. In addition, study results will be presented at national and international conferences in pediatric neurology, rehabilitation medicine, and assistive robotics. A plain-language summary of key findings will be prepared and shared with participating families and local rehabilitation centers. No participant-identifying information will be included in any reports or publications.

## 3 Discussion

Previous studies have primarily evaluated robotic-assisted gait training in ambulatory children with CP (GMFCS I–III) or have been limited to small-scale feasibility pilots (10, 12). To our knowledge, no long-term randomized controlled trial has targeted children classified as GMFCS level IV, a group typically excluded from intensive gait training due to perceived feasibility concerns. This protocol therefore addresses a substantial and underexplored need by proposing a structured, intensive gait training program tailored to children with minimal voluntary motor control.

This study leverages a state-of-the-art pediatric exoskeleton that integrates real-time motion sensing, adaptive feedback, and adjustable actuation. By combining mechanical support with task-specific gait practice and embedded motivational features (e.g., gamified feedback), the intervention aims to engage both motor and cognitive systems in children who are typically restricted to passive forms of therapy. The inclusion of individualized progression criteria and therapist supervision ensures that the intervention can be personalized while maintaining safety standards.

A key strength of the study lies in its multidimensional outcome framework. In addition to gross motor function and walking capacity, the trial will assess lower-limb joint range of motion, muscle morphology via ultrasound, and psychological satisfaction using a validated pediatric quality of life instrument. This broad set of measures acknowledges that motor disability in CP is multifaceted, with physical limitations often interlinked with emotional and social constraints. Furthermore, by including both child and caregiver-reported data, the study seeks to capture meaningful changes from a patient-centered perspective.

Certain limitations are acknowledged. The generalizability of results may be constrained to urban clinical settings with access to robotic technology and trained rehabilitation staff. In addition, while the sample size has been calculated to detect clinically meaningful changes, the trial may be underpowered for more exploratory subgroup analyses. Another limitation relates to the heterogeneity of the control group. Because “usual care” in China encompasses a wide spectrum of caregiver-directed and outpatient physiotherapy practices, comparisons with robotic gait training may appear imbalanced. However, this design choice reflects the ecological reality faced by families of children with GMFCS IV CP, where standardized rehabilitation is often unavailable. To mitigate this limitation, we will systematically document the type, frequency, and intensity of usual care and perform sensitivity analyses across subgroups, thereby ensuring transparency and interpretability of the findings. Furthermore, ankle ROM was selected as the sole joint-level outcome due to its clinical relevance and feasibility in children with GMFCS IV CP. We acknowledge that this choice does not capture potential deformities at the knee or hip. Future studies with expanded protocols should consider multi-joint assessments to provide a more comprehensive picture of musculoskeletal adaptations.

Finally, this trial does not include a formal cost-effectiveness analysis. Robotic-assisted gait training represents a new intervention, and its feasibility in non-ambulatory populations must be carefully weighed against healthcare resource constraints. At present, this technology is still being developed and pilot-tested in a limited number of research institutions and hospitals, and to our knowledge remains pending full registration within China’s medical device regulatory framework. Consequently, a formal economic evaluation at this stage is not possible. Nonetheless, potential benefits may extend beyond the restoration of walking ability. By reducing secondary complications such as contractures, musculoskeletal pain, and the need for orthopedic surgery, robotic training could eventually help offset long-term medical and caregiving costs. Moreover, improvements in quality of life and reductions in caregiver burden, though harder to quantify economically, carry important societal value. This study is therefore best viewed as an initial step to establish clinical feasibility and therapeutic relevance in an underserved population, providing a foundation for future research that will explicitly integrate cost-effectiveness and implementation science frameworks once the technology achieves broader regulatory approval and dissemination.

If effective, this study could establish preliminary evidence supporting the use of robotic gait assistance in a population that has long been underserved in rehabilitation research. It may also inform policy decisions regarding the broader integration of pediatric assistive robotics into community rehabilitation services in China and beyond. Ultimately, the findings will help determine whether high-tech, personalized interventions can feasibly enhance physical function and quality of life in children with severe CP.
